# Draft Genome Sequence of *Enterococcus casseliflavus* PAVET15 Obtained from the Oviduct Infection of the Cattle Tick (*Rhipicephalus microplus*) in Jiutepec, Morelos, Mexico

**DOI:** 10.1128/genomeA.00196-17

**Published:** 2017-04-20

**Authors:** R. Cossío-Bayúgar, E. Miranda-Miranda, C. A. Arreguín-Pérez, L. Lozano, D. Peréz de la Rosa, M. K. Rocha-Martínez, M. A. Bravo-Díaz, B. Sachman-Ruiz

**Affiliations:** a Centro Nacional de Investigación Disciplinaria en Parasitología Veterinaria del Instituto Nacional de Investigaciones Forestales, Agrícolas y Pecuarias, Morelos, Mexico; b Servicio Nacional de Sanidad, Inocuidad y Calidad Agroalimentaria, Morelos, Mexico; c Centro de Ciencias Genómicas, Universidad Nacional Autónoma de México, Cuernavaca, Morelos, Mexico

## Abstract

*Enterococcus* spp. are Gram-positive lactic acid-producing bacteria found in the intestinal tracts of animals, like mammals, birds, and arthropods. *Enterococcus* spp. may cause oportunistic infections in vertebrate and invertebrate hosts. We report here the draft genome sequence of *Enterococcus casseliflavus* PAVET15 containing 3,722,480 bp, with 80 contigs, an *N*_50_ of 179,476 bp, and 41.93% G+C content.

## GENOME ANNOUNCEMENT

Enterococcal bacteria are found in several ecosystems and are useful as a fecal indicator for water quality and models of infection involving core and variable genetics elements that are not fully understood (1). Bacteria belonging to the genus *Enterococcus* are part of the microbiome of the cattle tick gut and may be found in female, male, and egg samples (2); additionally, several isolates of *Enterococcus* species bacteria from actual infections in engorged female ticks were obtained (3, 4).

Here, we present the draft genome sequence of *Enterococcus casseliflavus* PAVET15, obtained from an engorged female tick that showed symptoms of bacterial infection, such as the presence of an exudate at the genital orifice. This strain was grown overnight in Trypticase soy agar (TSA; Difco-BD) medium at 37°C, and the genomic DNA was extracted using the Wizard genomic purification kit (Promega). The sequence genome was obtained using 454 FLX pyrosequencing (Roche) by building DNA fragment libraries, according to the manufacturer’s recommendations. The genome was assembled using Newbler version 2.8, obtaining a total of 3,722,480 bp, 80 contigs, an *N*_50_ of 179,476 bp, average contig size of 46,531 bp, and largest contig of 349,357 bp. Genome sequence annotation was made by RAST [http://rast.nmpdr.org (5)]. The resulting genome comprises 3,594 coding proteins, 58 rRNAs, 50 tRNAs, and 41.93% G+C content. We found 93.58% identity with *E. casseliflavus* and high synteny. For phylogenetic species differentiation, we used *rpoB* and *tufA* genes (data not shown). The virulent genes found in the strain PAVET15 could be relevant in the effort to find a biocontrol in the integral strategy to control the cattle tick.

### Accession number(s).

This whole-genome shotgun project has been deposited at DDBJ/ENA/GenBank under the accession no. MUBE00000000. The version described in this paper is version MUBE01000000.
